# American Medical Association

**Published:** 1854-04

**Authors:** 


### AMERICAN MEDICAL ASSOCIATION.

We would again call the attention of the profession of this State to the fact that the above named association will meet at St. Louis on the 2d May proximo. Our friends will also remember that an objection was raised to the appointment of a meeting at St. Louis, upon the supposition that the medical men of the West would not attend numerously. It was maintained on the other hand, that there was as much zeal in the medical public in the West as elsewhere, and that if an opportunity presented they would manifest it, provided an opportunity was afforded them without too heavy a sacrifice of time and means.

We have before urged the propriety of organizing Societies, and trust that the suggestion has been acted upon and that delegates have been chosen. We have not learned, however, that there has been any movement of the kind. We trust that Iowa, which can boast of as large a number of zealous and successful men as any of the Western States, in proportion to her population, will be well represented on that occasion. We shall see.

